# Suicides in Crete: frequency and gender/ seasonal distribution before and during the Covid-19 pandemic

**DOI:** 10.1192/j.eurpsy.2023.1691

**Published:** 2023-07-19

**Authors:** M. Basta, G. Karkanaki, E. Kranioti, A. Papadomanolakis, A. Kanaki, S. Belivanis, C. Lilitsis, E. Koutentaki, C. Panierakis, A. Vgontzas

**Affiliations:** 1 Psychiatry; 2 Forensic Medicine, university of Crete, School of Medicine; 3 Forensic Medicine, FORENSIC AGENCY OF CRETE, HERAKLION, CRETE; 4 Forensic Medicine, FORENSIC AGENCY OF CRETE, CHANIA CRETE, Greece

## Abstract

**Introduction:**

Covid pandemic-related psychological problems mainly include anxiety, depression and sleep disturbances, while evidence regarding suicidality is conflicting between studies. Crete has the highest suicide rate in Greece.

**Objectives:**

To examine suicides’ rate, gender and seasonal distribution pre-covid, compared to Covid-19 pandemic (2020-2021). Seasonality was studied by quarter of the year.

**Methods:**

Data on the number of suicides, demographics, and seasonal distribution by quarter throughout Crete, were collected from the records of the Department of Forensic Medicine/University Hospital of Heraklion Crete, as well as from the Forensic Agency of Crete

**Results:**

We found that in the years 2020 and 2021, number of suicides in Crete are 41 and 40 respectively and do not differ from those of the previous years (Mean_1999-2019_ = 43.5/year). Also, the distribution of suicides by gender remained stable (Mean men/women _1999-2019_=4.6 vs. Mean men/women _2020-2021_=4.3). The analysis of the seasonal distribution of suicides showed a reversal of the seasonal distribution in 2020 compared to the previous 5-years, which tends to return to pre-pandemic characteristics in 2021.

**Conclusions:**

The overall rate and gender distribution of suicides in Crete remained stable during the Covid pandemic compared to the previous twenty years. The clear change in the seasonal distribution of suicides in 2020 compared to the distribution of previous years tends to return to previous levels in 2021. Possibly, the change in seasonality in 2020 is related to the fear of death/stress and the short/medium term economic consequences due to the pandemic.

**Disclosure of Interest:**

None Declared

